# The Usage of AI in Teaching and Students’ Creativity: The Mediating Role of Learning Engagement and the Moderating Role of AI Literacy

**DOI:** 10.3390/bs15050587

**Published:** 2025-04-27

**Authors:** Min Zhou, Song Peng

**Affiliations:** College of Physical Education, Sichuan University, Chengdu 610065, China; zm_scu@scu.edu.cn

**Keywords:** the usage of AI in teaching, creativity, learning engagement, resource conservation of resources theory, AI literacy

## Abstract

With the rapid development of Artificial Intelligence (AI) technology, the application of AI in the field of education has gradually become one of the key factors in improving teaching quality and student abilities. Based on the conservation of resources theory, this study explores how the usage of AI in teaching impacts students’ creativity, exploring the mediating role of learning engagement and the moderating role of AI literacy. The research finds that the usage of AI in teaching significantly enhances students’ creativity, with learning engagement playing a mediating role in this process, thereby promoting creativity improvement. In addition, AI literacy moderates the relationship between the usage of AI in teaching and learning engagement. The results of this study not only expand the application of the conservation of resources theory in the field of education but also emphasize the important role of AI literacy in AI teaching, providing valuable policy suggestions for educational practices.

## 1. Introduction

In today’s era of ubiquitous digital and intelligent education, the effective use of artificial intelligence (AI) technology by teachers in the classroom is not only an important way to drive innovation in educational models, but also a key method for advancing the professionalization, personalization, and intelligence of learning (Celik et al., 2022; Wu et al., 2024). For the purpose of this study, AI refers to technologies and systems that simulate human intelligence, including tools that can analyze data, adapt to students’ learning needs, provide personalized feedback, and support the automation of educational tasks. The usage of AI in teaching can provide personalized teaching plans for teachers, improve teaching efficiency, and tailor typical exercises and feedback mechanisms according to student characteristics, thereby maximizing the training of students’ thinking abilities and creativity (Ng et al., 2023). Therefore, studying the impact of the usage of AI in teaching on various aspects of students, particularly focusing on the specific mechanisms related to students’ creativity, holds significant research value (Fahimirad & Kotamjani, 2018).

Creativity, as a key competency increasingly emphasized in both international and domestic educational contexts, refers to students’ ability to generate creative ideas and solutions within the course of their learning (Jahnke et al., 2017). This ability is critical in the process of applying knowledge in innovative ways, which contributes to novel outputs and problem-solving in real-world scenarios (Richardson & Mishra, 2018). The teaching methods, tools, and approaches employed by educators play a pivotal role in nurturing students’ creative potential. AI technology, in particular, not only alleviates teachers from repetitive tasks but also provides diverse and personalized learning structures that align with students’ individual characteristics, effectively fostering their ability to explore creative thinking and generate novel ideas (Rong et al., 2022). Therefore, investigating the mechanisms through which AI usage in teaching influences students’ ability to produce creative ideas is of significant practical and theoretical importance.

The conservation of resources theory suggests that people expend a large number of resources when focusing and engaging (Westman et al., 2004). Learning engagement is an important indicator of the intrinsic motivation and external investment students exhibit during the learning process (Halverson & Graham, 2019). Teachers’ effective use of AI technology, through personalized feedback, intelligent evaluation, and active learning incentives, can maximize students’ resource investment and drive them to complete more creative tasks (Ke et al., 2016). Therefore, this study demonstrates the mediating role of learning engagement in the relationship between the usage of AI in teaching and students’ creativity.

Based on the conservation of resources theory, teachers’ AI literacy is considered an important professional resource that influences how teachers engage with and utilize resources in the classroom (Halbesleben et al., 2014). In the context of education, AI literacy refers to the competencies that enable teachers to critically evaluate AI technologies, collaborate with AI, and use AI tools to enhance teaching and learning in various settings, including classrooms and online environments (Long & Magerko, 2020). This includes an understanding of AI’s potential and limitations, integrating AI into pedagogical practices, and fostering students’ own AI literacy and critical thinking. Teachers with high AI literacy are able to understand and effectively use AI tools in teaching and assessments, address teaching needs promptly, and maintain high levels of engagement (Long & Magerko, 2020). However, in this study, we shift the focus from measuring teachers’ self-reported AI literacy to exploring students’ perceptions of their teachers’ AI literacy. While teachers may assess their own AI literacy, students’ perceptions provide a different perspective, capturing how they observe and experience AI integration in the classroom. These perceptions are critical, as they reflect students’ experiences with AI-enhanced learning environments and how teachers’ use of AI influences classroom interaction and learning efficiency. Students’ awareness of how AI is used by their teachers can impact their own creativity, engagement, and learning outcomes (Ng et al., 2021). Therefore, in this study, we examine students’ perceptions of teachers’ AI literacy as a moderating factor in how AI technology in teaching influences students’ creativity. This approach underscores the importance of students’ perceptions in understanding the effectiveness and impact of AI in education. It should be noted that the AI literacy mentioned later refers to the teacher’s AI literacy perceived by students.

In summary, this study explores how the usage of AI in teaching impacts students’ creativity, exploring the mediating role of learning engagement and the moderating role of AI literacy.

## 2. Hypothesis Development

### 2.1. The Usage of AI in Teaching and Student Creativity

Based on the conservation of resources theory, the enhancement in students’ creativity through the usage of AI in teaching can be understood through the mechanisms of resource accumulation and reinvestment (Hobfoll & Shirom, 2000). First, the introduction of AI technology helps teachers efficiently handle tedious teaching tasks, such as lesson planning, grading, and student behavior analysis, thus saving a significant amount of time and effort (Singh & Hiran, 2022; Wu et al., 2025). This enables teachers to devote more energy to teaching innovation and the personalized development of students, increasing their resource reserves (such as time, knowledge, and energy), which provides the possibility for further educational reform (Al Darayseh, 2023).

Next, teachers convert these surplus resources into specific teaching advantages through AI technology (Kaplan-Rakowski et al., 2023). For instance, AI can provide personalized learning content based on students’ learning progress and interests, design open-ended questions and project-based tasks, help students engage in deep thinking, and stimulate their creative thinking (Zimmerman, 2018). Additionally, AI can track students’ learning states in real time, offering personalized feedback that fosters students’ reflection and innovation (Ali, 2020). However, some studies have raised concerns that AI-driven tutoring systems may lead to students’ over-reliance on technology, potentially diminishing their critical thinking and independent knowledge acquisition abilities (J. Sengewald et al., 2024; Van Slyke et al., 2023). If students excessively depend on AI for answers and guidance, they may struggle to develop problem-solving skills and the ability to evaluate information critically. Therefore, while AI offers significant benefits in education, it is crucial that we implement it in a way that encourages students’ autonomy and cognitive engagement rather than the passive consumption of information (Kshirsagar et al., 2022).

Finally, the AI-powered teaching environment allows students to accumulate a variety of resources, including information, knowledge, and skills, during their diversified and personalized learning process (Ogunleye et al., 2024; Wu et al., 2024). This accumulation of resources not only enhances students’ ability to solve complex problems but also provides ample support for their creativity development (Balacheff, 1993). At the same time, students’ creative achievements provide feedback to teachers, boosting teachers’ sense of accomplishment and confidence, which motivates them to invest more in innovative teaching practices. This creates a positive cycle of resource accumulation and reinvestment between teachers and students, promoting the continuous enhancement in students’ creativity (Nazaretsky et al., 2022). Therefore, this study proposes the following hypothesis:

**H1.**  *The usage of AI in teaching positively influences students’ creativity.*

### 2.2. The Mediating Role of Learning Engagement

The conservation of resources theory suggests that individuals tend to acquire, maintain, and protect resources that help them cope with stress and promote goal achievement (Hobfoll & Shirom, 2000; Ke et al., 2016; Li et al., 2024). In the teaching environment, teachers’ introduction of AI technology can provide students with rich learning resources, flexible learning methods, and personalized learning support, thereby enhancing students’ learning engagement (Noe et al., 2010). For instance, AI-driven intelligent tutoring systems can offer customized feedback and suggestions based on students’ learning progress and needs, stimulate students’ interest in learning, and maintain their learning motivation (Chan et al., 2021; Noe et al., 2010). Furthermore, virtual learning environments and AI-generated interactive content can capture students’ attention, increasing interaction and participation during the learning process (Chen, 2017). Therefore, the usage of AI in teaching can enhance learning resources’ availability and effectiveness, thereby promoting students’ learning engagement.

Learning engagement typically includes behavioral, emotional, and cognitive dimensions, reflected in students’ active involvement, emotional resonance, and deep thinking during learning activities (J. Lee et al., 2022). The conservation of resources theory emphasizes that, when individuals feel that resources are sufficient and supportive, they are more likely to invest energy in exploration and innovation (Li et al., 2025; Martin & Borup, 2022). A high level of learning engagement encourages students to continuously stimulate curiosity and the desire for exploration, thus fostering the development of creativity (Nkhoma et al., 2014). For example, students actively participating in classroom discussions, project-based learning, and problem-solving activities are more likely to propose novel ideas and innovative solutions (Ertmer & Newby, 1993). Additionally, behavioral engagement is manifested through students’ active participation in classroom activities, emotional engagement is reflected in their interest in learning content, and cognitive engagement is shown through the in-depth understanding of and reflection on learning content (Appleton et al., 2008). Therefore, learning engagement, as a bridge for students to invest in learning and acquire new knowledge, significantly enhances students’ creativity.

Based on the conservation of resources theory, the usage of AI in teaching, by increasing learning resources, improving learning support, and promoting interaction, can effectively improve students’ learning engagement (M. Wang & Kang, 2006). For example, AI technology can identify students’ weak learning areas through big data analysis and provide targeted supplementary resources; virtual reality (VR) technology can create an immersive learning environment, increasing the fun and interactivity of learning (Conrad & Openo, 2018). Meanwhile, the enhancement in learning engagement further stimulates students’ creativity. Therefore, learning engagement plays a key mediating role in the relationship between the usage of AI in teaching and students’ creativity (Mercer, 2019). Based on this, the study proposes the following hypothesis:

**H2.**  *Students’ learning engagement mediates the relationship between the usage of AI in teaching and students’ creativity.*

### 2.3. The Moderating Role of AI Literacy

Based on the conservation of resources theory, AI literacy plays an important moderating role in the relationship between the usage of AI in teaching and students’ learning engagement. However, in this study, we specifically focus on students’ perceptions of teachers’ AI literacy, which reflects how students observe and experience their teachers’ use of AI technology in the classroom. Students’ perceptions provide an external viewpoint on how effectively AI is integrated into the teaching process and how it influences their learning engagement.

When students perceive that their teachers have higher AI literacy, they are likely to notice that teachers can efficiently integrate AI tools into their teaching practices. These teachers are perceived as being able to understand and effectively use AI technology to personalize learning content, automate assessments, and provide real-time feedback (Druga et al., 2019). Such perceptions of high AI literacy by students suggest that teachers are reducing the cognitive load and minimizing the time consumption caused by technical barriers, thus freeing up more resources for instructional design and fostering positive teacher–student interactions (Southworth et al., 2023). According to the conservation of resources theory, when teachers are perceived by students as effectively utilizing external resources like AI, this helps conserve and expand the internal resources available for teaching, thus improving the learning experience (Hobfoll & Shirom, 2000).

In turn, students are more likely to engage actively in learning activities when they perceive their teachers as being proficient with AI technology. This increased learning engagement results from students perceiving their teachers as capable of optimizing their learning experience with AI tools, which can lead to more personalized and efficient learning opportunities (Almatrafi et al., 2024). On the other hand, if students perceive their teachers as lacking AI literacy or find AI tools difficult to use, they may resist engaging with AI, thus hindering their learning engagement (T. Sengewald & Tremmel, 2024). Teachers who are perceived to have high AI literacy are better able to address these concerns by demonstrating the benefits of AI and making it accessible and understandable to students (Kim et al., 2020).

Additionally, when students perceive their teachers as confident and competent in using AI technology, this often translates into positive teaching behaviors and attitudes in the classroom. These perceptions can foster an environment that stimulates students’ enthusiasm and initiative to learn (I. Lee et al., 2021). In contrast, if students perceive their teachers as having low AI literacy, they may observe difficulties in the teacher’s use of technology, which can disrupt the flow of the teaching process, increase the cognitive load, and reduce overall teaching effectiveness (Kong et al., 2024). This negative perception of AI use can lead to lower learning engagement, as students may feel less motivated to participate in learning activities that are hindered by technical difficulties.

Thus, in the context of students’ perceptions, teachers’ AI literacy acts as a moderating factor that can either strengthen or weaken the positive impact of AI on students’ learning engagement. If students perceive their teachers as proficient with AI, the positive effects on learning engagement are more likely to be realized. Conversely, if students perceive their teachers as struggling with AI, this may diminish the potential benefits of AI integration in the classroom. Hence, the study proposes the following hypothesis:

**H3.**  *AI literacy moderates the relationship between the usage of AI in teaching and students’ learning engagement.*

The conservation of resources theory emphasizes that individuals tend to acquire, maintain, and protect resources that help them cope with stress and achieve goals (Hobfoll & Shirom, 2000). In the teaching context, learning engagement, which refers to the psychological and behavioral resources that students invest in learning activities, is a crucial factor in promoting creativity development (Ng et al., 2022). At the same time, the use of AI in teaching provides students with abundant resources, enhances learning engagement, and, ultimately, fosters creativity development (Hong & Kim, 2024; Ng et al., 2022).

However, in this study, we specifically focus on how students perceive their teachers’ AI literacy and how this perception impacts their learning engagement. When students perceive their teachers as having high AI literacy, they are more likely to observe the efficient use of AI tools that optimize teaching resources, provide real-time feedback, and offer personalized guidance, which significantly increases students’ learning engagement (Bewersdorff et al., 2025). From the perspective of the conservation of resources theory, when students perceive that their teachers effectively manage AI resources, it enhances their sense of support and learning effectiveness, thereby strengthening the mediating role of learning engagement in the relationship between AI use in teaching and students’ creativity (Hobfoll & Shirom, 2000). In this case, students’ perceptions of high AI literacy amplify the mediating role of learning engagement, meaning that the use of AI in teaching, as perceived by students, more significantly promotes the development of students’ creativity through increased learning engagement (Eguchi et al., 2021).

In contrast, when students perceive their teachers as having low AI literacy, they may notice inefficiencies in the use of AI tools, such as delayed feedback, resource misallocation, or the ineffective integration of AI, which can reduce teaching effectiveness. According to the conservation of resources theory, such perceptions of resource depletion or mismanagement may trigger stress responses, leading to a decline in students’ learning engagement (Park & Yi, 2021). When students feel unsupported or disengaged due to these issues, it weakens the mediating role of learning engagement between AI use and creativity (Mertala et al., 2022). Therefore, when students perceive low AI literacy in their teachers, the positive impact of AI use on creativity through learning engagement is diminished, as the mediating effect of engagement is weakened. Thus, the study proposes the following hypothesis:

**H4.**  *AI literacy moderates the mediating role of students’ learning engagement between the usage of AI in teaching and creativity. When AI literacy is high, the mediating role of learning engagement is stronger.*

**Figure 1:**
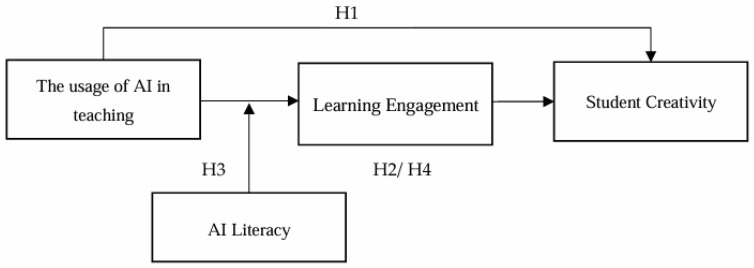
Research model.

In summary, the research model is shown in Figure 1.

## 3. Methods

### 3.1. Participants and Sampling

The participants in this study consisted of students from the universities in Sichuan Province, with the goal of exploring how the usage of AI in teaching influences students’ creativity, as well as examining the mediating role of learning engagement and the moderating role of AI literacy. A stratified random sampling method was employed to select participants from students with different regional and disciplinary backgrounds to ensure the sample’s representativeness. The selection criteria for participants included the following: (1) participants must be enrolled students whose courses involve AI-assisted teaching (e.g., teachers using AI tools for explanations, providing learning resources, or personalized feedback); (2) participants must have basic knowledge of AI to ensure they understand and can answer questions related to AI in the survey; and (3) students must voluntarily participate in the study and maintain a relatively stable learning environment during the data collection period. Ultimately, 432 valid questionnaires were collected, covering both male and female students from various age groups.

In this study, the participants were exclusively students from universities in Sichuan Province. The survey was administered to these students, who were asked to evaluate their teachers’ AI literacy based on their perceptions of teachers’ use of AI tools in the classroom. The variable of AI literacy was, therefore, measured from the students’ perspective, rather than directly assessing the teachers’ own literacy. Additionally, the study measured student creativity and engagement through self-reported data provided by the students themselves. This allowed us to explore how students’ perceptions of teachers’ AI literacy influence their own creativity, learning engagement, and overall educational experience.

The data collection process was conducted in two stages. The first stage, conducted in September 2024, focused on investigating the usage of AI in teaching, AI literacy, and other control variables to ensure participants met the selection criteria. The second stage, conducted in October 2024, focused on collecting data related to students’ learning engagement and creativity, aiming to examine the subsequent impact of the usage of AI in teaching on students’ creativity. Data were collected using a combination of online platforms and offline questionnaires, with all participants voluntarily participating and providing informed consent before completing the survey.

### 3.2. Measurements

The variables in this study were measured using well-established scales from both domestic and international sources. Foreign scales were translated and back-translated to ensure clarity and consistency. To ensure the applicability of these scales within the Chinese context, two Ph.D. students reviewed the scales. All questionnaires used a 5-point Likert scale, where 1 represented “strongly disagree” and 5 represented “strongly agree”. Please refer to Appendix A for specific scale items.

The usage of AI in teaching: The variable was measured using a scale developed by Medcof (1996). The internal consistency coefficient of this variable was 0.94.

Learning engagement: The Learning Engagement scale developed by Dixson (2015) was used to measure this variable. The internal consistency coefficient for this variable was 0.89.

AI literacy: To measure teachers’ AI literacy, we adapted the AI Literacy scale developed by B. Wang et al. (2023), which was originally designed for self-assessment, to assess students’ perceptions of their teachers’ AI literacy. We believe that students’ perspectives are crucial, as teachers’ self-assessments may not always reflect the actual experience and AI application level perceived by students in the classroom. This adaptation shifts the focus from self-reported competence to students’ observations of how teachers use AI in their teaching. The internal consistency coefficient for this variable was 0.85. Given the adaptation of the AI Literacy scale to measure students’ perceptions of teachers’ AI literacy, we conducted additional analyses to ensure the quality and validity of the modified instrument. First, we performed a confirmatory factor analysis (CFA) to assess the factor structure and construct validity of the adapted scale. The results indicated a good fit, confirming that the revised scale effectively captures the intended constructs related to students’ perceptions of teachers’ AI literacy. Additionally, we calculated the internal consistency coefficient (Cronbach’s alpha) for the adapted scale, which was 0.85, demonstrating strong reliability. To further validate the scale, we also examined its discriminant and convergent validity, and the results supported the validity of the instrument in capturing students’ perceptions. These supplementary analyses provide strong evidence that the modified AI Literacy scale is a valid and reliable measure of students’ perceptions of teachers’ AI literacy, making it suitable for use in this study.

Student creativity: The scale developed by Snyder et al. (2019) was used to measure students’ creativity. The internal consistency coefficient for this variable was 0.96.

Control variables: Demographic variables, such as age and gender, were used as control variables. Age was measured using an open-ended question where participants directly provided their age, and gender was measured with a multiple-choice question where participants selected their gender.

### 3.3. Data Analysis Strategy

For data analysis, the study employed statistical software such as SPSS 25.0, Mplus 7.4, and AMOS 24.0. First, data cleaning was performed to remove invalid questionnaires, followed by descriptive statistical analysis. Next, hierarchical regression analysis was used to test the hypotheses, with the Bootstrap method applied to examine the mediating effects. Interaction term regression analysis was used to verify the moderating effects. This structured data analysis approach ensured the scientific and reliable nature of the data, providing strong empirical support for understanding how the usage of AI in teaching affects students’ creativity.

## 4. Results

### 4.1. Common Method Bias Test

To ensure the validity of our results and address potential concerns regarding common method bias (CMB), we conducted a test for common method bias. CMB refers to the systematic variance shared among the measures of different constructs due to the method used to collect data, rather than the constructs themselves. If not addressed, CMB can distort the true relationships between variables and lead to spurious conclusions. In this study, we used Harman’s single-factor test to check for CMB, which is a widely accepted method in the social sciences. The results of this test help ensure that the observed relationships between the constructs in our study are not primarily driven by a single underlying factor, but, rather, reflect genuine associations between the variables. By performing this test, we aim to enhance the robustness and validity of our findings.

To check for common method bias, we used Harman’s single-factor test. An exploratory factor analysis was conducted without rotation for all the items of the variables. The result indicated that the first principal component had an eigenvalue greater than 1 and explained 21.34% of the variance. This was well below the 40% threshold, suggesting that common method bias was not a significant issue in this study. Additionally, since Harman’s single-factor test might not be very sensitive, we added an error variable factor to the five-factor model. We then compared this model with the original five-factor model, finding that the changes in the model fit indices were minimal (CFI = 0.022, ΔTLI = 0.002, and ΔRMSEA = 0.006). This further supports the conclusion that common method bias is not a significant concern in this study.

### 4.2. Descriptive Statistical Analysis

The means, standard deviations, and correlation coefficients of the variables involved in this study are shown in Table 1. From Table 1, we can observe the following significant correlations: the usage of AI in teaching is positively correlated with AI literacy (r = 0.17, *p* < 0.001), learning engagement (r = 0.28, *p* < 0.001), and student creativity (r = 0.23, *p* < 0.01). Learning engagement is positively correlated with student creativity (r = 0.28, *p* < 0.001). These correlations provide preliminary support for the hypotheses presented in this study.

### 4.3. Confirmatory Factor Analysis

To ensure the validity and reliability of the constructs in our study, we performed Confirmatory Factor Analysis (CFA). CFA is a statistical technique used to test whether a set of observed variables (i.e., items in a survey or scale) adequately represents a smaller number of latent constructs (i.e., unobserved theoretical variables). In the context of this study, CFA allows us to confirm that the measurement model, which links the observed variables to the underlying constructs, is consistent with the theoretical framework. This process is crucial for ensuring that each construct—such as AI literacy and student creativity—is measured accurately, and that the relationships between the observed items align with the theoretical expectations. By conducting CFA, we assess the fit of the proposed model and ensure the constructs are measured reliably, providing a solid foundation for subsequent analyses.

In this study, CFA was conducted using Mplus 7.4. First, a four-factor model (baseline model) was constructed. Then, three-factor, two-factor, and single-factor models were constructed for comparison, with the results shown in Table 2. After comparing the fit indices of each model, we found that the four-factor model (baseline model) showed significantly better fit indices than the other models (*χ*^2^/*df* = 2.22, RMSEA = 0.06, CFI = 0.96, TLI = 0.94), indicating that the discriminant validity between the main variables is good, and the research data passed the confirmatory factor analysis test.

### 4.4. Hypothesis Testing

**Figure 2:**
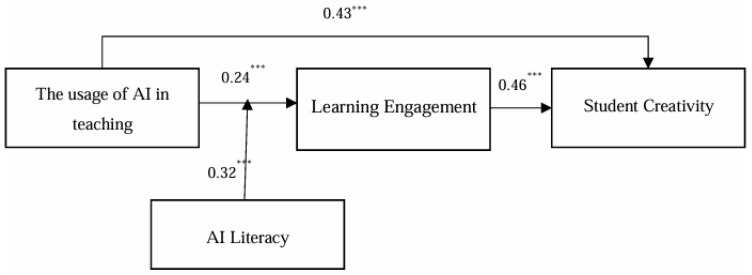
The result of hypothesis test. (*** *p* < 0.001).

In this study, structural equation modeling (SEM) was conducted using Mplus 7.4 to test the hypotheses. The path coefficients and their significance are shown in Figure 2.

As shown in Figure 2, the usage of AI in teaching significantly and positively affects learning engagement (B = 0.24, *p* < 0.001) and student creativity (B = 0.43, *p* < 0.001). Therefore, H1 is supported. Additionally, learning engagement significantly and positively influences student creativity (B = 0.46, *p* < 0.001).

To test the mediation effect of learning engagement between the usage of AI in teaching and student creativity, the Preacher method with conditional indirect effects (Bootstrap = 5000) was used (Preacher & Hayes, 2008). The results indicate that learning engagement plays a significant mediation role, with an indirect effect value of 0.25. The 95% confidence interval for the indirect effect is [0.23, 0.34], which does not include 0, confirming that the mediation effect is valid. Hence, H2 is supported.

**Figure 3:**
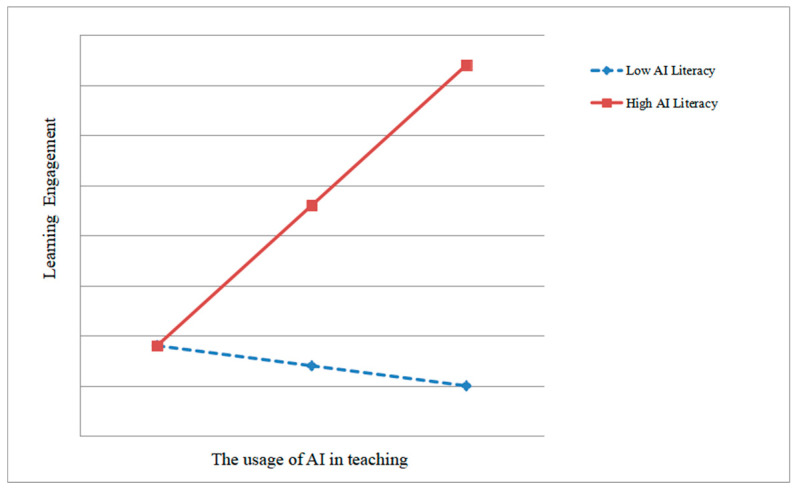
The moderate role of AI literacy.

As shown in Figure 2, AI literacy moderates the relationship between the usage of AI in teaching and learning engagement (interaction term coefficient B = 0.32, *p* < 0.001). Thus, H4 is supported. To further verify H4, the simple slope method was used to plot the moderation effect of AI literacy on the relationship between the usage of AI in teaching and learning engagement, as shown in Figure 3.

The Bootstrap method was used to randomly sample 5000 times to test the moderated mediation effect, and the results are presented in Table 3. According to Table 3, when AI literacy is low, the mediation effect of learning engagement is 0.23, with a 95% confidence interval of [0.07, 0.29], not including 0. When AI literacy is high, the mediation effect is 0.15, with a 95% confidence interval of [−0.14, 0.19], which includes 0. These results indicate a significant difference. Therefore, H4 is confirmed.

## 5. Discussion

### 5.1. Theoretical Contributions

First, this study expands the research perspective on the impact of the usage of AI in teaching on students’ creativity. Existing studies mainly focus on the application of AI technology in education concerning learning efficiency and academic performance, while research on creativity, a higher-order cognitive ability, remains relatively limited (Long & Magerko, 2020; Martin & Borup, 2022; Mercer, 2019; Nazaretsky et al., 2022; Snyder et al., 2019). By examining creativity development, this study reveals how the usage of AI in teaching promotes students’ creativity. It provides a new theoretical perspective for interdisciplinary research between artificial intelligence and creativity education and offers theoretical support for future explorations into how AI technology fosters creative thinking.

Second, this study identifies the mediating role of learning engagement between the usage of AI in teaching and creativity. While existing research emphasizes the potential of AI technology in education, the specific mechanisms underlying its effects remain underexplored (Long & Magerko, 2020; Mercer, 2019; Ng et al., 2023; Ng et al., 2022). By introducing learning engagement as a mediator, this study confirms that the usage of AI in teaching does not directly influence creativity but rather enhances students’ creativity by increasing their engagement and initiative. This finding deepens our understanding of how the usage of AI in teaching impacts students’ creative development and enriches the application of learning engagement theory in AI education. It also provides new insights into the pathways through which AI affects learning outcomes (Li et al., 2023; Wu et al., 2023).

Third, this study reveals the moderating role of teachers’ AI literacy in the effectiveness of AI-assisted teaching. It finds that teachers’ AI literacy influences the extent to which the usage of AI in teaching enhances students’ learning engagement and creativity. This expands the theoretical discussion on how educators’ characteristics affect the effectiveness of technology-enhanced education (Mertala et al., 2022; Noe et al., 2010; Richardson & Mishra, 2018). This contribution not only reinforces the theoretical value of teachers’ AI literacy in educational settings but also introduces a new moderating variable perspective for future research. It highlights the crucial role of teachers’ competencies in the usage of AI in teaching (Rong et al., 2022; Southworth et al., 2023; M. Wang & Kang, 2006). Moreover, this finding has important implications for educational practice, emphasizing that promoting teachers’ AI literacy is a key factor in the successful integration of AI into education.

### 5.2. Practical Contributions

First, the study confirms that the usage of AI in teaching effectively enhances students’ creativity, demonstrating the immense potential of AI in education. Therefore, educational institutions and teachers should actively explore AI applications in the classroom. For instance, AI-generated content (AIGC) tools can support students in creative writing, design, and artistic creation, while intelligent tutoring systems can provide personalized learning recommendations. Additionally, teachers should continuously improve their understanding and application of AI technology—not only as users of AI tools but also as facilitators who guide students in leveraging AI for creative thinking. Schools and policymakers should provide relevant training and resources to help teachers effectively integrate AI into their teaching practices, maximizing AI’s role in fostering students’ creativity.

Second, learning engagement serves as a bridge between AI-assisted teaching and student creativity. This suggests that simply using AI technology does not automatically enhance creativity; rather, AI’s positive effects depend on increasing students’ learning engagement. Therefore, educators should design learning environments that encourage active student participation. For example, AI-driven virtual experiments, gamified learning platforms, and personalized learning pathways can allow students to explore and create through hands-on experiences, thus enhancing their interest and focus. Additionally, educators should prioritize students’ learning experiences by combining AI technology with interactive teaching methods such as group discussions and project-based learning (PBL), which can foster students’ active knowledge construction and indirectly promote creativity. For policymakers, it is crucial that we emphasize the integration of AI technology in education while also strengthening AI literacy to maximize its positive impact on creativity development. Policymakers can focus on providing training and support for schools and teachers, helping them effectively utilize AI technology while enhancing student engagement. Such initiatives would not only improve teaching quality but also cultivate students’ innovation skills, preparing them for future challenges.

Third, students’ perceptions of their teachers’ AI literacy significantly influence the extent to which AI usage in teaching enhances student engagement and creativity. This highlights that the way students perceive teachers’ understanding and ability to use AI plays a crucial role in determining the effectiveness of AI-assisted teaching outcomes. Therefore, schools and policymakers should prioritize improving students’ perceptions of their teachers’ AI literacy by supporting teachers’ professional development in AI skills. Training programs that include workshops and courses on AI tools, programming, machine learning, and data literacy can help teachers enhance their AI capabilities, which, in turn, can positively influence how students perceive and engage with AI in the classroom. Additionally, schools should encourage teachers to explore both the potential and limitations of AI, allowing them to design pedagogical strategies that effectively integrate AI tools into teaching. For teachers who are perceived by students as highly AI-literate, institutions should foster opportunities for them to experiment with advanced AI applications to innovate teaching methods further. For those teachers who are perceived as having lower AI literacy, targeted training and hands-on experiences should be provided to help reduce technological barriers and increase students’ confidence in the use of AI. By addressing these areas, schools can ensure that students perceive their teachers as proficient with AI, which will maximize the benefits of AI-assisted teaching and its impact on student engagement and creativity.

### 5.3. Research Limitations and Future Directions

First, this study may be limited by the sample selection, as the participants were primarily students from specific regions and educational stages. This may affect the generalizability of the findings. Future research should expand the sample scope to include students from different countries, educational levels (e.g., primary school, and secondary school), and disciplines to verify the robustness of the findings and explore the variations in AI-assisted teaching effects across different educational contexts.

Second, this study primarily employs a cross-sectional data analysis, making it difficult to precisely capture causal relationships, particularly the long-term effects of AI-assisted teaching on students’ creativity. Future research could adopt longitudinal study designs to track students’ long-term performance in AI-supported learning environments or employ experimental and intervention studies to more accurately verify the mechanisms of the usage of AI in teaching effects on creativity. Additionally, incorporating qualitative methods such as interviews and classroom observations could provide deeper insights into how the usage of AI in teaching influences students’ learning experiences and creative development.

Third, this study treats the usage of AI in teaching as a broad concept. However, AI technology in education takes many forms, such as intelligent tutoring systems, AI-generated content (AIGC), and personalized recommendation systems, each of which may have distinct effects on student creativity. Future research could further differentiate between these AI applications and examine their specific impacts in various teaching contexts. Additionally, future studies could explore how teachers can effectively integrate AI technology into teaching practices to optimize instructional strategies and enhance students’ creativity and other core competencies.

One potential limitation of this study is the composition of the sample. The participants were students who were already familiar with AI, which may have influenced their attitudes toward the technology. Prior research suggests that students’ perceptions of AI’s usefulness and ease of communication play a key role in their willingness to adopt it (Kim et al., 2020). Since our sample consisted of students who had prior exposure to AI, they may have been more receptive to its integration into learning activities. This could limit the generalizability of our findings to students who are less familiar with AI or who may hold skeptical views about its role in education. Future studies should consider including a more diverse sample, including students with varying levels of AI exposure and attitudes, to better understand the broader implications of AI adoption in education.

## 6. Conclusions

This study explores the impact of the usage of AI in teaching on students’ creativity and reveals the mediating role of learning engagement as well as the moderating role of AI literacy. The findings indicate that the usage of AI in teaching not only directly enhances students’ creativity but also exerts an indirect effect by increasing learning engagement. Additionally, teachers’ AI literacy plays a crucial moderating role in this process. Based on these findings, educators should focus on designing AI-enhanced learning environments that promote student engagement and creativity, while policymakers should prioritize initiatives that support AI literacy development for both teachers and students to optimize AI’s impact on creativity. Future research could further investigate the specific effects of different types of AI tools in educational settings, as well as other potential influencing mechanisms, to provide more comprehensive theoretical support and practical guidance for AI-powered education.

## Figures and Tables

**Table 1:** Means, standard deviations, correlations, and reliability among study variables.

Variable	Mean	Mode	SD	1	2	3	4	5	6	7
1 Gender	1.25	1	0.44	1						
2 Age	20.22	23	3.34	0.013	1					
3 AI Literacy	3.64	3	1.63	0.28	0.35	1				
4 Learning Engagement	4.14	4	1.48	0.46	0.23	0.28 **	1			
5 Student Creativity	2.31	3	0.86	−0.23	0.13 *	0.34 **	0.28 ***	1		
6 The usage of AI	4.12	4	1.23	0.12	0.03	0.17 **	0.28 **	0.23 **	1	

Note: * *p* < 0.05; ** *p* < 0.01; *** *p* < 0.001.

**Table 2:** Confirmatory factor analysis.

Model	*χ* ^2^ */df*	CFI	TLI	RMSEA
Four-factor model	2.22	0.96	0.94	0.06
Three-factor model	6.97	0.74	0.66	0.12
Two-factor model	10.23	0.72	0.51	0.16
One-factor model	13.43	0.44	0.41	0.20

**Table 3:** Moderated mediation effect test results.

Variable	Effect	SE	Lower Limit of 95% Confidence Interval	Higher Limit of 95% Confidence Interval
Mean − 1 SD	0.23	0.03	0.07	0.29
Mean + 1 SD	0.15	0.33	−0.14	0.19

## Data Availability

The data that support the findings of this study are available from the first author upon reasonable request. We were unable to share the data due to the participant’s request.

## Appendix A

Items of the usage of AI in teaching
  1. My teacher frequently used AI tools to carry out most teaching tasks.
  2. Most of the class time involved my teacher working with AI tools.
  3. My teacher used AI tools to support major teaching decisions.
Items of Learning Engagement
  1. Making sure to study on a regular basis
  2. Putting forth effort
  3. Staying up on the readings
  4. Looking over class notes between getting class to make sure I understand the material
  5. Being organized
  6. Taking good notes over readings, PowerPoints, or video lectures
  7. Listening/reading carefully
  8. Finding ways to make the course material relevant to my life
  9. Applying course material to my life
  10. Finding ways to make the course interesting to me
  11. Really desiring to learn the material
  12. Having fun in chats, discussions with the instructor or other students
  13. Participating actively in small-group discussion forums
  14. Helping fellow students
  15. Getting a good grade
  16. Doing well on the tests/quizzes
  17. Engaging in conversations
  18. Posting in the discussion forum regularly
  19. Getting to know other students in the class
Items of Student Creativity
  1. I have generated innovative solutions or ideas during my studies that have helped me solve complex problems or challenges in my coursework.
  2. I have applied concepts or skills learned in class to create new ideas, projects, or solutions outside of the classroom setting.
  3. I have produced original work (e.g., essays, presentations, or projects) in my coursework that was recognized for its creativity by peers or instructors.
  4. I have contributed creative ideas or solutions during group work or collaborative projects, enhancing the overall outcome of the project.
  5. I have used the knowledge and skills gained in my education to develop creative solutions to real-world problems or challenges, demonstrating practical application of learning.
Items of AI Literacy
  1. My teacher can distinguish between smart devices and non-smart devices.
  2. My teacher knows how AI technology can help in teaching and learning.
  3. My teacher can identify the AI technology used in the educational tools and platforms they utilize.
  4. My teacher can skillfully use AI applications or products to support their teaching and enhance student learning.
  5. It is usually hard for my teacher to learn to use a new AI application or product.
  6. My teacher uses AI applications or products to improve teaching efficiency and effectiveness.
  7. My teacher can evaluate the capabilities and limitations of an AI application or product after using it for a while.
  8. My teacher can choose a proper solution from various AI-driven tools or platforms available.
  9. My teacher can select the most appropriate AI application or product for specific tasks in the classroom.
  10. My teacher always complies with ethical principles when using AI applications or products in their teaching.
  11. My teacher is always aware of privacy and information security issues when using AI applications or products.
  12. My teacher is always alert to the potential abuse of AI technology in the classroom.
